# Dietary supplementation of heat-treated *Gracilaria* and *Ulva* seaweeds enhanced acute hypoxia tolerance in gilthead sea bream (*Sparus aurata*)

**DOI:** 10.1242/bio.024299

**Published:** 2017-05-11

**Authors:** Leonardo J. Magnoni, Juan Antonio Martos-Sitcha, Augusto Queiroz, Josep Alvar Calduch-Giner, José Fernando Magalhães Gonçalves, Cristina M. R. Rocha, Helena T. Abreu, Johan W. Schrama, Rodrigo O. A. Ozorio, Jaume Pérez-Sánchez

**Affiliations:** 1 CIIMAR, University of Porto, Terminal de Cruzeiros do Porto de Leixões, Av. General Norton de Matos s/n, Matosinhos 4450-208, Portugal; 2 IIB-INTECH, Av. Intendente Marino Km. 8.2, Chascomús 7310, Argentina; 3 Nutrigenomics and Fish Growth Endocrinology Group, Institute of Aquaculture Torre de la Sal (CSIC), Ribera de Cabanes, Castellón 12595, Spain; 4 ICBAS, University of Porto, Rua de Jorge Viterbo Ferreira n.° 228, Porto 4050-313, Portugal; 5 REQUIMTE, LAQV, Departamento de Engenharia Química, Faculdade de Engenharia, Universidade do Porto, Rua Dr. Roberto Frias, Porto 4200-465, Portugal; 6 ALGAplus, Lda., Travessa Alexandre da Conceição S/N, Ílhavo 3830-196, Portugal; 7 Aquaculture and Fisheries group, WIAS, Wageningen University, AH Wageningen 6700, The Netherlands

**Keywords:** Hypoxia, Oxidative stress, Sea bream, Nutritional background, Seaweeds

## Abstract

Intensive aquaculture practices involve rearing fish at high densities. In these conditions, fish may be exposed to suboptimal dissolved O_2_ levels with an increased formation of reactive O_2_ species (ROS) in tissues. Seaweeds (SW) contain biologically active substances with efficient antioxidant capacities. This study evaluated the effects of dietary supplementation of heat-treated SW (5% *Gracilaria vermiculophylla* or 5% *Ulva lactuca*) on stress bioindicators in sea bream subjected to a hypoxic challenge. 168 fish (104.5 g average weight) were distributed in 24 tanks, in which eight tanks were fed one of three experimental diets for 34 days: (i) a control diet without SW supplementation, (ii) a control diet supplemented with *Ulva*, or (iii) a control diet with *Gracilaria*. Thereafter, fish from 12 tanks (*n*=4 tanks/dietary treatment) were subjected to 24 h hypoxia (1.3 mg O_2_ l^−1^) and subsequent recovery normoxia (8.6 mg O_2_ l^−1^). Hypoxic fish showed an increase in hematocrit values regardless of dietary treatment. Dietary modulation of the O_2_-carrying capacity was conspicuous during recovery, as fish fed SW supplemented diets displayed significantly higher haemoglobin concentration than fish fed the control diet. After the challenge, survival rates in both groups of fish fed SW were higher, which was consistent with a decrease in hepatic lipid peroxidation in these groups. Furthermore, the hepatic antioxidant enzyme activities were modulated differently by changes in environmental O_2_ condition, particularly in sea bream fed the *Gracilaria* diet. After being subjected to hypoxia, the gene expression of antioxidant enzymes and molecular chaperones in liver and heart were down regulated in sea bream fed SW diets. This study suggests that the antioxidant properties of heat-treated SW may have a protective role against oxidative stress. The nature of these compounds and possible mechanisms implied are currently being investigated.

## Abstract (precis)

**Summary:** The physiological response and survival rate of gilthead sea bream to hypoxia was highly modulated by dietary supplementation of heat-treated seaweed.

## INTRODUCTION

All aerobic organisms rely on the presence of O_2_ to obtain energy via oxidative phosphorylation (OXPHOS) in the mitochondria. Low environmental O_2_ (hypoxia) represents a major physiological challenge, in which cells have to switch energy generation from OXPHOS to anaerobic glycolysis (Barbour and Turner, 2014). Mitochondria are not only the major O_2_ consumers within the cells, but are also known to be major producers of reactive O_2_ species (ROS) (Murphy, 2009). Electron leaking from complex I and III from the electron transport chain (ETC) in mitochondria are important sources of ROS formation (Görlach et al., 2015). In addition to the ETC, there are several other ROS-producing sites in mitochondria, such as the enzymes pyruvate dehydrogenase and α-ketoglutarate dehydrogenase (Quinlan et al., 2014; Starkov et al., 2004). High ROS levels will generate oxidative stress, which may result in accumulative oxidative damage to DNA, RNA, proteins and lipids, and may invoke profound functional changes (Birben et al., 2012). Fish contain multiple antioxidant systems to counteract the deleterious effects of ROS (Lushchak, 2011). The antioxidant defence system is formed by substances such as vitamins C and E, glutathione and carotenoids, together with several enzymes capable of reducing ROS or oxidized products. In particular, enzymatic activities in fish, including catalase (CAT), glutathione peroxidase (GPX) and glutathione reductase (GR), are known to be modulated by nutritional and environmental conditions (Martínez-Álvarez et al., 2005). In addition to that, a number of transcriptional factors are among the ROS targets, responding positively or negatively to nutrients and to environmental cues by altering gene expression. One of the most well-characterized transcriptional factors in mammalian systems is the hypoxia-inducible factor-1 (HIF-1), which appears to integrate the responses to different primary stimuli at the level of ROS signalling (Semenza and Wang, 1992). However, it remains unclear how acute changes in environmental O_2_ condition may induce signals for transcriptional regulation of cell functions in aquatic species, and, most importantly, how this response could be modulated by nutritional factors.

Seaweeds (SW) are important marine sources of polysaccharides and the main industrial application is in the hydrocolloids (agar) industry. However, the agar industry generates large amounts of solid by-products, which are often discharged. Solid waste agar extraction from SW source may exhibit various biological activities, including anti-oxidant or anti-tumoral properties that are of interest in livestock production. Furthermore, the heat treatment during agar extraction was shown to produce additional antioxidant compounds in the SW by-product (Yoshiki et al., 2009), including an increase in polyphenol content (Rajauria et al., 2010). Recently, the number of studies on utilization of SW by-product as a natural source of functional ingredients has been growing rapidly (Kumar et al., 2008; Thirunavukkarasu et al., 2013). In fact, SW have been frequently associated with health benefits due to the radical scavenging and singlet O_2_ quenching activity present in dry, raw, and cooked preparations (Kumar and Brown, 2013; Sachindra et al., 2010). The presence of antioxidant compounds in SW have been suggested as an endogenous defence mechanism protecting against oxidative stress due to extreme environmental conditions (Aguilera et al., 2002). Dietary supplementation of phytochemicals in animal feed may enhance not only antioxidant capacities, but also may act as a low-dose chronic stressor, preparing the cells to resist to severe stress (Speciale et al., 2011). Red SW, such as *Gracilaria vermiculophylla*, are characterized by their pigments, including chlorophyll a, carotenoids, phycobilins (Cardoso et al., 2014), halogenated compounds (Amsler, 2008), and polyphenols with antioxidant activity (Duan et al., 2006; Yuan et al., 2005; Yuan and Walsh, 2006), which make this group of SW promising supplements in aquatic feeds (Holdt and Kraan, 2011). Dietary supplementation with *G. vermiculophylla* and several SW alters the metabolic and antioxidant responses without compromising the growth of the European seabass (*Dicentrarchus labrax*) (Peixoto et al., 2016a,b). Studies in white shrimp *Litopenaeus vannamei* fed a diet supplemented with *G. vermiculophylla* suggested a modulatory effect on the antioxidant capacities when animals are subjected to biotic and abiotic stressors (Chen et al., 2012; Sirirustananun et al., 2011). Additionally, the green SW *Ulva lactuca* has been shown to have potent antioxidant effects when tested in several mammalian experimental models, which may be related to the presence of polysaccharides (Hassan et al., 2011; Qi et al., 2005; Wang et al., 2014), phenols or flavonoids (Farasat et al., 2014), or a combination of all those compounds (Mezghani et al., 2013). Thus, dietary inclusion of 5% *U. lactuca* had beneficial effects on growth performance in the white spotted snapper (*Lutjanus stellatus*), although high levels (20%) of *U. lactuca* may produce hepatic damage (Zhu et al., 2016).

The gilthead sea bream (*Sparus aurata*) is an opportunistic feeder that consumes SW as part of its natural diet (Arias, 1980; Pita et al., 2002), being an important species in terms of total aquaculture production in southern Europe. Moreover, *S. aurata* has become a valuable animal model on nutritional and environmental studies. Cultured fish species in floating sea cages may be exposed to low O_2_ levels (Remen et al., 2015). In addition, Remen et al. (2015) has shown in sea bream that the limiting O_2_ saturation value (LOS, also termed as Pcrit) is approximately 2.3 mg O_2_ l^−1^ at 19°C, which is within the average range of Pcrit value reported by Rogers et al. (2016) for other marine species. *S. aurata* increases hepatic antioxidant enzyme activities when subjected to hypoxia (Pérez-Jiménez et al., 2012b), and modulates the hepatic expression of several genes involved in β-oxidation, oxidative stress response, and energy generation in mitochondria in response to different dietary lipid sources (Pérez-Sánchez et al., 2013; Saera-Vila et al., 2009). Also, the oxidative status of sea bream is improved upon dietary supplementation with essential amino acids when fed plant protein-based diets (Sitjà-Bobadilla et al., 2005) or when antioxidants, such as methionine and white tea, are supplied in the diet (Pérez-Jiménez et al., 2012a).

The current study evaluated the effects of dietary supplementation with heat-treated *G. vermiculophyll*a or *U. lactuca*, followed by acute hypoxia and subsequent recovery, on the metabolic profile and antioxidant capacity in sea bream juveniles. Additionally, the transcriptional level of selected markers associated with oxidative stress and mitochondrial activity were evaluated in liver and heart. Both are target tissues in studies on energy use and redox balance during hypoxia (Everett et al., 2012; Hermes-Lima et al., 2001).

## RESULTS

### Fish performance

Zootechnical parameters during the feeding trial are presented in Table 1. No significant differences as a result of dietary treatment were observed for any of the parameters analysed.

**Table 1.**
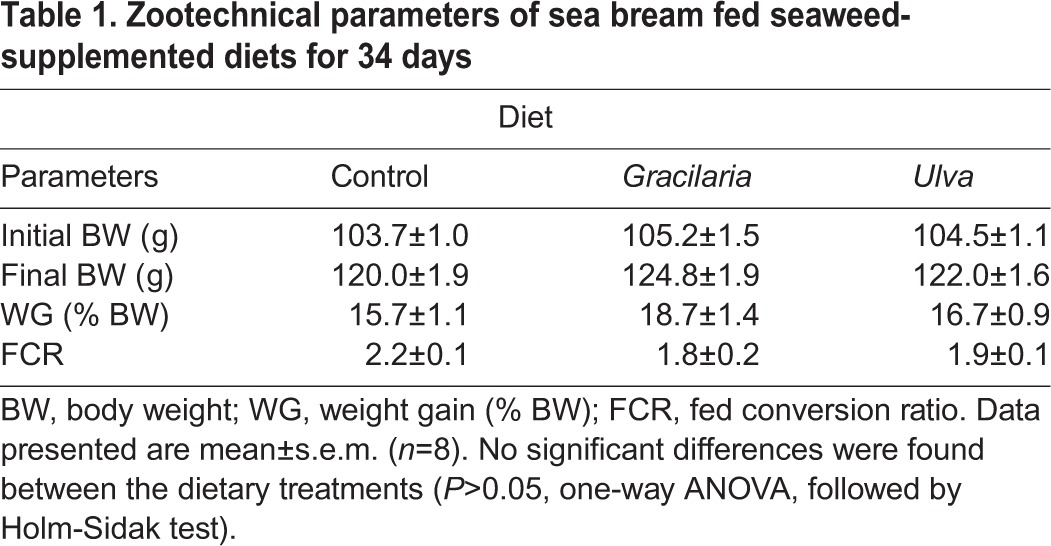
**Zootechnical parameters of sea bream fed seaweed- supplemented diets for 34 days**

### Accumulative mortality during hypoxia and recovery

**Fig. 1.**
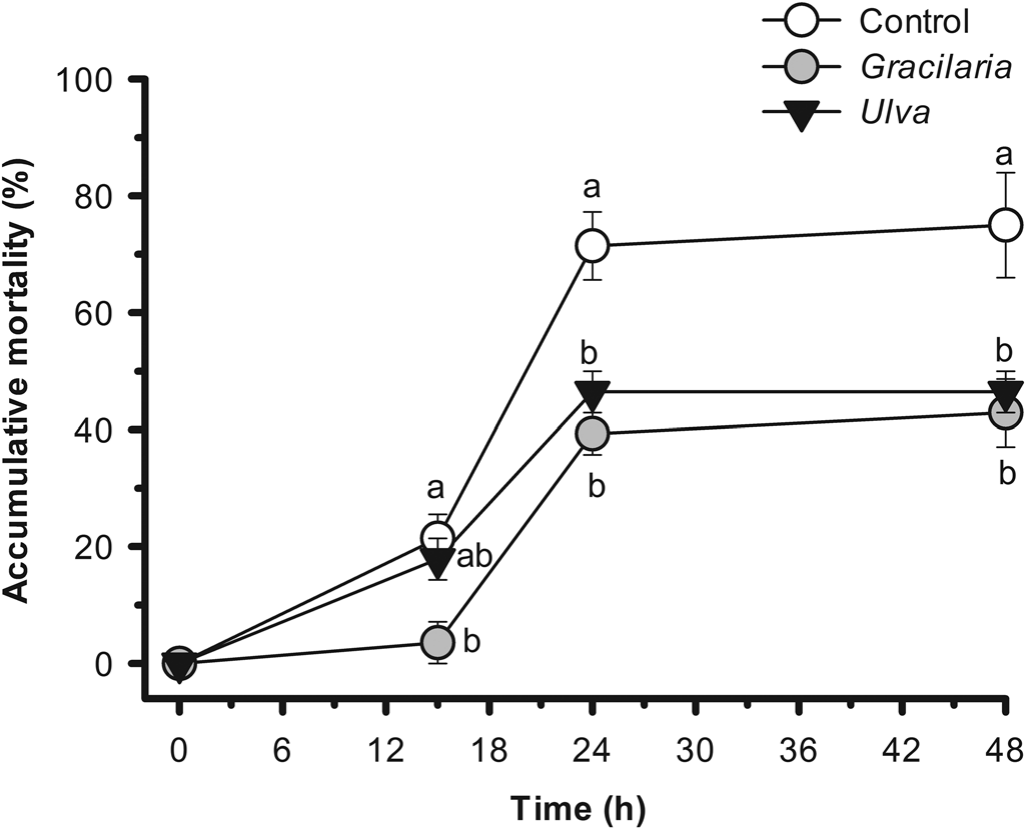
**Changes in accumulative mortality of sea bream fed seaweed- supplemented diets and subjected to hypoxia followed by normoxia (recovery).** Data are represented as mean±s.e.m. of four tanks per treatment. Bars with different letters indicate significant differences between dietary treatments (*P*<0.05, one-way ANOVA, followed by Holm-Sidak test).

The accumulative mortality observed in sea bream subjected to hypoxia and subsequent recovery differed between dietary treatments (Fig. 1). 15 h after subjecting the animals to hypoxia, the accumulative mortality was significantly higher in sea bream fed the control diet than in those fed the *Gracilaria* diet [*P*<0.05, 21.4±4.1 and 3.6±3.6%, respectively (mean±s.e.m.)]. This differential mortality between dietary treatments became more marked at the end of the hypoxia challenge. After 24 h of hypoxia, the accumulative mortality of fish fed the control diet was significantly higher than in sea bream fed both *Gracilaria* and *Ulva* diets (*P*<0.05, 71.4±5.8, 39.3±3.6, and 46.4±3.6%, respectively).

### Blood and plasmatic parameters

Changes in selected blood and plasma parameters as an effect of diet and environmental O_2_ condition are presented in Table 2. No significant differences were observed in the hematocrit (Hct), haemoglobin (Hb), or mean corpuscular Hb concentration (MCHC) values between fish fed different diets. Fish showed changes in the values of the three blood parameters when subjected to changes in environmental O_2_ condition, but no interaction with the dietary treatment was detected. Fish subjected to hypoxia showed significant higher Hct values than in normoxia (*P*<0.01). Nevertheless, in contrast to the *Gracilaria* group, the Hct in fish fed the control or *Ulva* diets was significantly reduced at recovery (*P*<0.05). On the other hand, haemoglobin (Hb) concentration remained unchanged in fish fed SW-supplemented diets, although showed a significantly increase in hypoxia in fish fed control diet (*P*<0.05). Also, in fish fed control diet, Hb concentration at recovery showed lower values than at normoxia (*P*<0.05). Regarding changes in MCHC, the response of the group fed *Ulva* to fluctuations in environmental O_2_ condition was distinct from the other dietary treatments, as the value for this parameter was lower at hypoxia (*P*<0.05), showing a partial recuperation during the recovery.

**Table 2.**
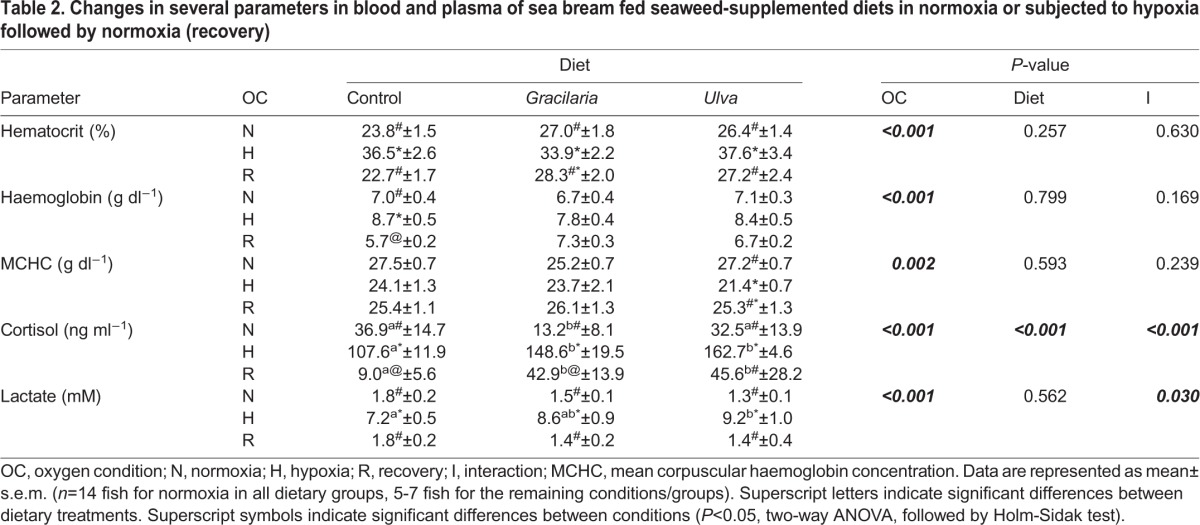
**Changes in several parameters in blood and plasma of sea bream fed seaweed-supplemented diets in normoxia or subjected to hypoxia followed by normoxia (recovery)**

Environmental O_2_ condition and diet significantly affected cortisol levels, also displaying interaction (*P*<0.01). In normoxia, cortisol levels were significantly lower in fish fed *Gracilaria* diet when compared to the other dietary treatments (*P*<0.05). Fish subjected to hypoxia showed increased cortisol in all the dietary treatments, displaying a significant decline on the level of this hormone at recovery (*P*<0.01). However, during hypoxia and recovery, fish fed *Gracilaria* or *Ulva* diets showed significantly higher cortisol levels than these fed the control diet (*P*<0.01).

Environmental O_2_ condition, but not diet, showed a significant effect on lactate. An interaction between environmental O_2_ condition and diet was detected. The concentration of plasma lactate was significantly higher at hypoxia than at normoxia or recovery for all the dietary treatments (*P*<0.01, four- to sevenfold-increases/decreases). In hypoxia, fish fed *Ulva* diet showed significantly higher lactate than fish fed the control diet (*P*<0.01).

### Hepatic oxidative stress markers

The effects of SW supplementation and environmental O_2_ condition on selected hepatic oxidative stress markers are presented in Table 3. Both environmental and dietary factors significantly altered lipid peroxidation (LPO) (*P*<0.001 and =0.01, respectively), also displaying interaction (*P*<0.01). In particular, LPO was significantly higher during hypoxia for all dietary treatments (*P*<0.05). However, LPO activity was differentially modulated by dietary treatments during the recovery period, showing the highest values in fish fed the control diet when compared to fish fed SW-supplemented diets (*P*<0.01).

**Table 3.**
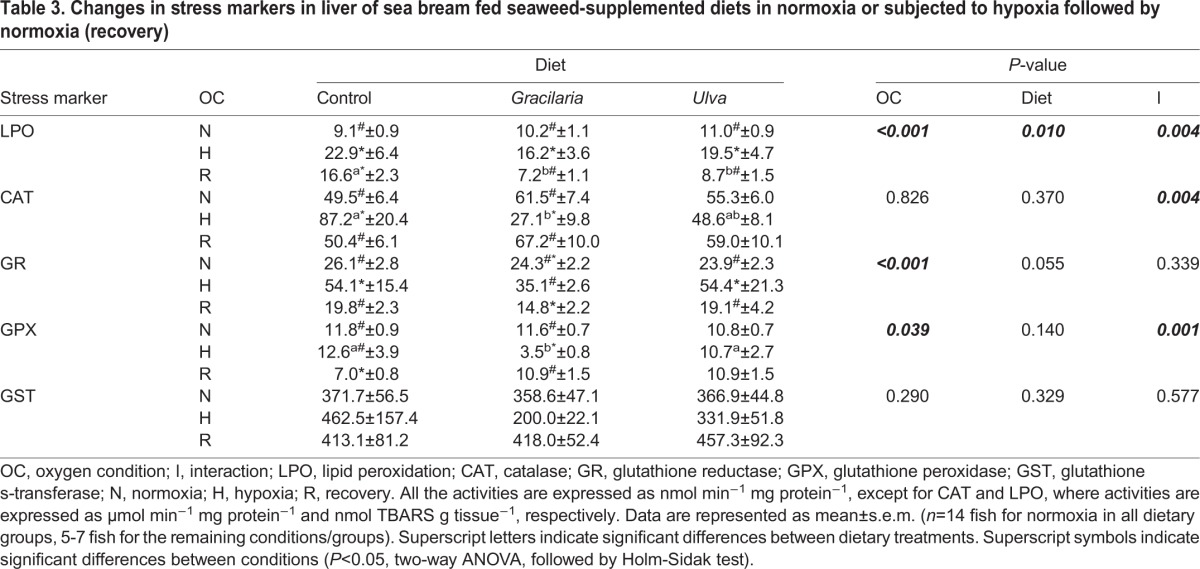
**Changes in stress markers in liver of sea bream fed seaweed-supplemented diets in normoxia or subjected to hypoxia followed by normoxia (recovery)**

Neither environmental nor dietary factors significantly altered catalase (CAT) activity, although an interaction between both factors was detected (*P*<0.01). Changes in CAT activity were noticeable in fish fed the control and *Gracilaria* diets, as the activity for this enzyme during hypoxia increased or decreased, respectively (*P*<0.05). A significant lower CAT activity was measured in hypoxic fish fed *Gracilaria* diet when compared to the control diet (*P*<0.05). Conversely, changes in CAT activity were less pronounced in fish fed *Ulva* diet.

Environmental O_2_ condition, but not diet, significantly altered GR activity (*P*<0.001), and no interaction was detected. Hypoxia produced an increase, whereas recovery induced a decrease of GR activity, regardless of the dietary treatment (*P*<0.001). However, GR activity in fish fed *Gracilaria* showed a sharper decrease during recovery with values even below than in normoxia (*P*<0.05). Changes in GPX activity with different environmental O_2_ condition were less conspicuous in all dietary groups (*P*<0.05). Diet did not affect GPX activity, although an interaction with environmental O_2_ condition was detected (*P*<0.01). A significant decrease in GPX activity was observed in fish fed *Gracilaria* diet during hypoxia (*P*<0.01). The GPX activity was increased at recovery on fish fed *Gracilaria* diet, whereas the opposite response was observed on those fish fed control diet. No significant differences in GST activity was detected in fish fed different diets or subjected to changes in environmental O_2_ condition. No interaction was detected for GST activity.

### Gene expression analyses in liver and heart

**Fig. 2.**
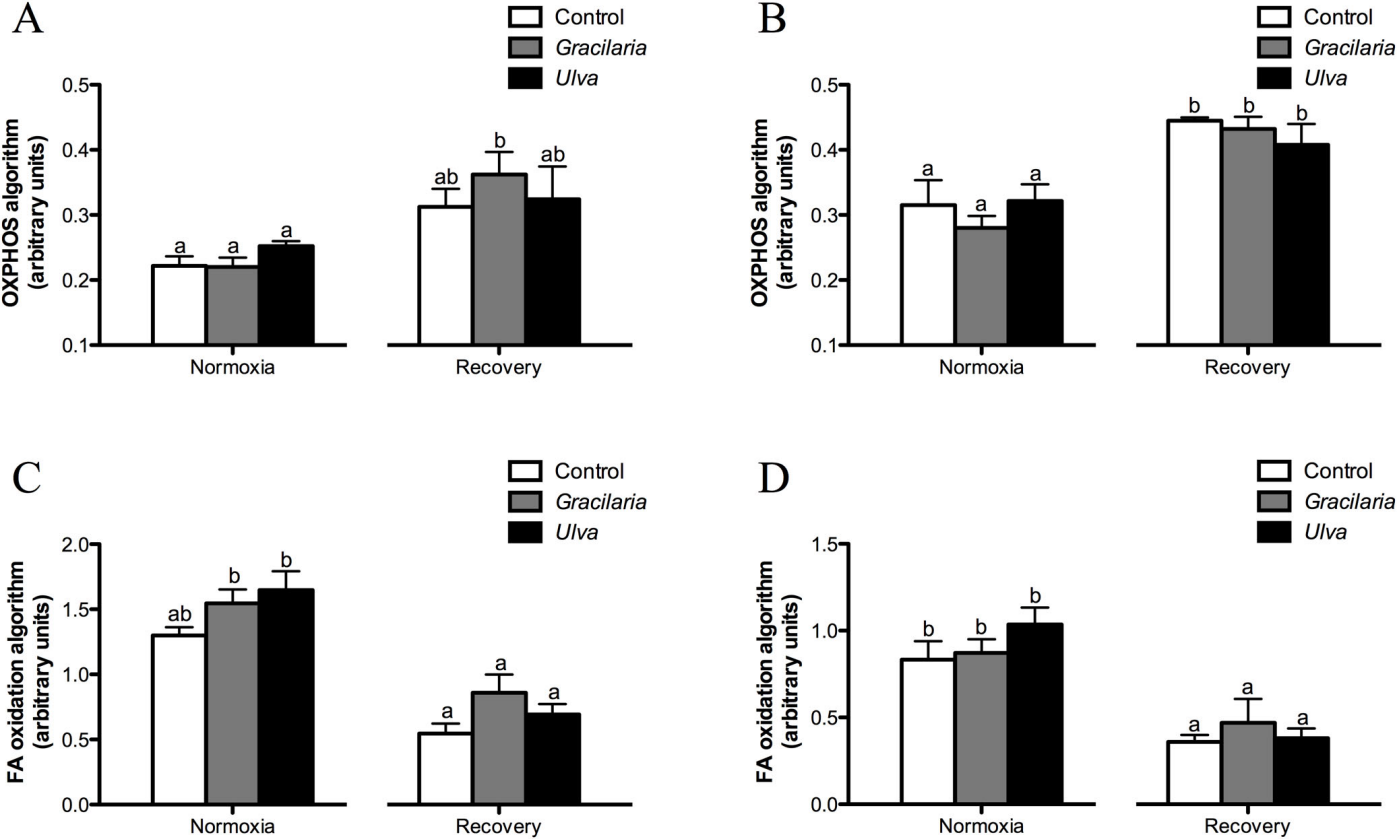
**Hepatic and cardiac indexes related to OXPHOS and FA oxidation genes in sea bream fed seaweed supplemented diets in normoxia or subjected to hypoxia followed by normoxia (recovery).** (A,C) Hepatic; (B,D) cardiac. Data are represented as mean±s.e.m. (*n*=5-7). Bars with different letters indicate significant differences between dietary treatments (*P*<0.05, one-way ANOVA, followed by Holm-Sidak test).

Data on hepatic and cardiac gene expression in fish fed the dietary treatments under normoxia and recovery are presented in Tables S2-S5. The calculated values for both OXPHOS and FA oxidation algorithms did not reveal any dietary effects, but importantly the OXPHOS index was consistently lower in normoxia than in recovery fish in both tissues (Fig. 2A,B). The opposite pattern was reported for the CPT1A/CS algorithm (Fig. 2C,D).

**Fig. 3.**
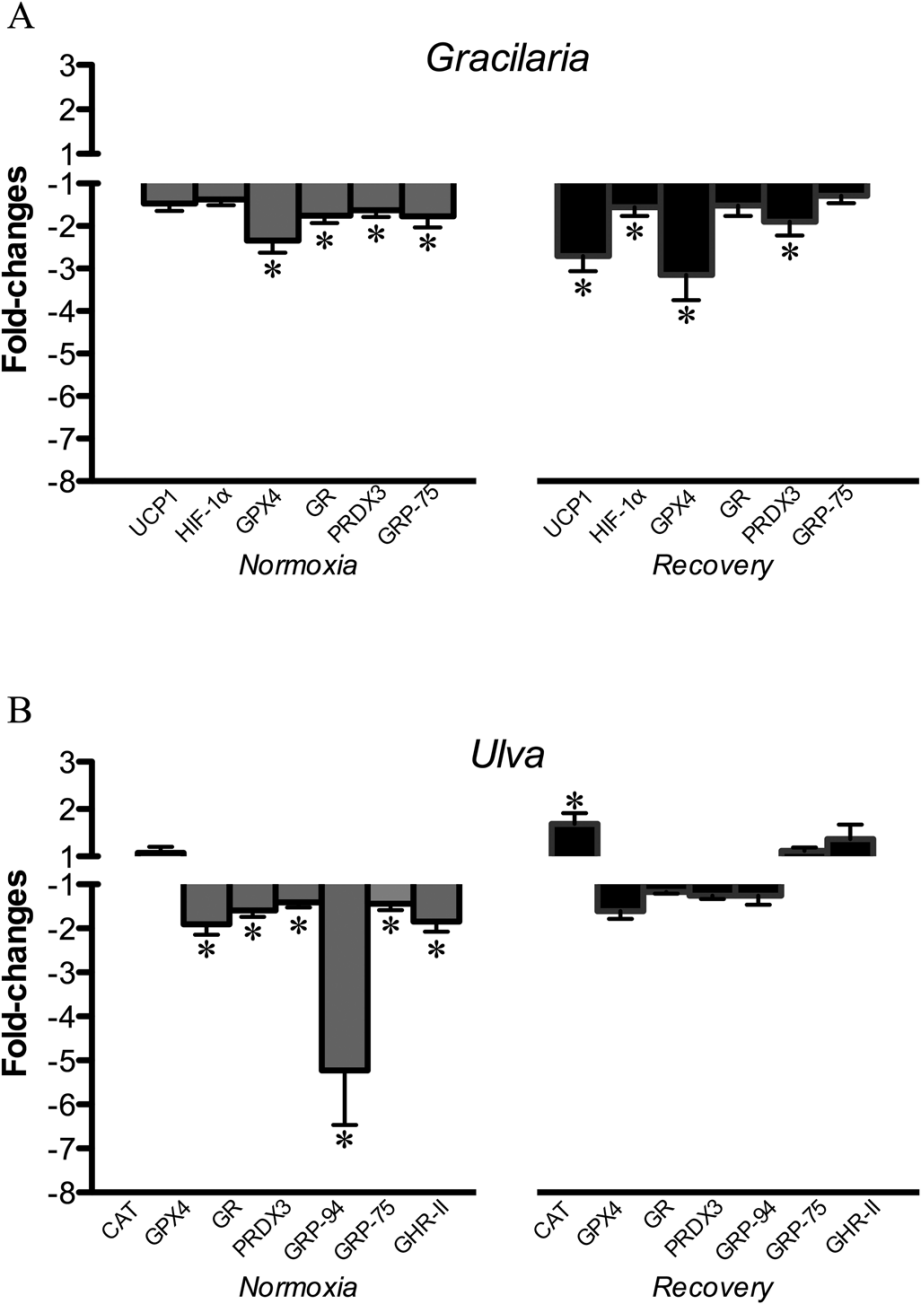
**Fold-changes of mRNA expression levels of differentially expressed genes in liver of sea bream fed seaweed-supplemented diets and subjected to normoxia or to hypoxia followed by normoxia (recovery).** (A) *Gracilaria* versus control; (B) *Ulva* versus control. Data are represented as mean±s.e.m. (*n*=5-7). Asterisks indicate significant differences with respect to the control diet group within each environmental O_2_ condition (*P*<0.05, Student *t*-test).

Gene by gene, up to six genes were differentially expressed in liver under normoxia or recovery when *Gracilaria* and control treatments were compared (Fig. 3A). The expression profile for *Gracilaria* fish under normoxia was a significantly down-regulated response of antioxidant defence enzymes (GPX4, GR, and PRDX3) and molecular chaperones (GRP-75). During recovery, the down-regulated response was maintained for GPX4 and PRDX3, including HIF-1α and uncoupling protein 1 (UCP1). A similar pattern was observed in fish fed *Ulva* diet (Fig. 3B), and the expression of six genes associated to antioxidant defence (GPX4, GR, PRDX3), molecular chaperones (GRP-94 and GRP-75), and GH/IGF axis (GHR-II) were down-regulated under normoxic conditions. Among them, GPX4, GR, PRDX3 and GRP-75 were identified as coincident down-regulated genes in fish fed *Gracilaria* and *Ulva* diets under normoxic conditions. Conversely, only one gene involved in CAT was up-regulated during recovery in fish fed the *Ulva* diet. Therefore, the magnitude (1 gene instead of 4) and the direction of change (up-regulation versus down-regulation) were highly influenced by the dietary treatment during recovery.

**Fig. 4.**
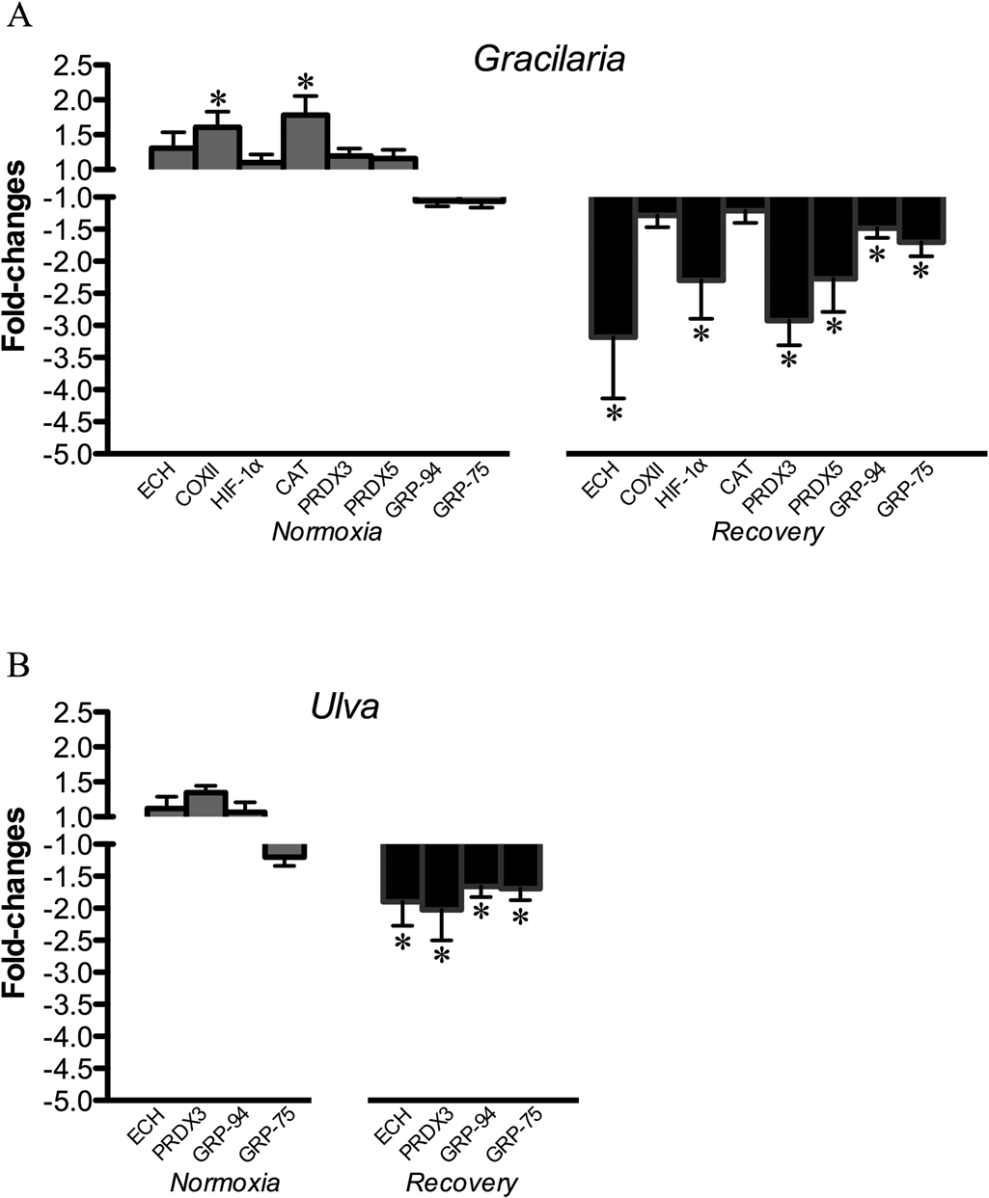
**Fold-changes of mRNA expression levels of differentially expressed genes in heart of sea bream fed seaweed-supplemented diets and subjected to normoxia or to hypoxia followed by normoxia (recovery)**. (A) *Gracilaria* versus control; (B) *Ulva* versus control. Data are represented as mean±s.e.m. (*n*=5-7). Asterisks indicate significant differences with respect to the control diet group within each environmental O_2_ condition (*P*<0.05, Student *t*-test).

In heart, *Gracilaria-*supplemented diet triggered the up-regulation of OXPHOS markers (COXII) and antioxidant enzymes (CAT) under normoxic conditions. Conversely, during recovery, *Gracilaria*-supplemented diet down-regulated the expression of HIF-1α, oxidative enzymes (ECH), antioxidant enzymes (PRDX3, PRDX5) and molecular chaperones (GRP-94, GRP-75) (Fig. 4A). In fish fed the *Ulva* diet, changes in gene expression were only observed in the recovery group with a significant down-regulation of ECH, PRDX3, GRP-94 and GRP-75 (Fig. 4B). These genes were recognized as common regulated genes in fish fed both *Gracilaria* and *Ulva* diets during recovery.

## DISCUSSION

Fish in intensive aquaculture practices are exposed to environmentally stressful conditions, particularly to fluctuations in O_2_ availability, as the activity involves rearing animals at high density. This study evaluated the role of dietary supplementation of heat-treated SW on the metabolic profile and antioxidant capacity in sea bream juveniles, during and after an acute hypoxic event. A wide range of commercial products may be utilized to the aqua-feeds industry if it is confirmed that the antioxidant defences of fish are mediated by heat-treated SW.

### Physiological parameters analysed in blood and plasma of sea bream confirmed the effectiveness of the hypoxic challenge

In the present study, sea bream exposed to a severe hypoxia (1.3 mg O_2_ l^−1^) for 24 h responded by increasing the Hct. This change indicated that sea bream boosted its O_2_-carrying capacity during hypoxic condition, in a similar manner as observed in eels and rainbow trout (Soivio et al., 1980; Wood and Johansen, 1972). Such enhancement in O_2_-carrying capacity may be caused by a swelling, release and/or formation of erythrocytes, and plasma volume reduction (Gallaugher and Farrell, 1998). Nevertheless, changes observed in the current study due to the hypoxic condition could be explained by erythrocyte swelling alone (Jensen et al., 1993), as Hb concentration and MCHC remained similar to the normoxic values. Surprisingly, dietary modulation of the O_2_-carrying capacity was conspicuous during recovery, but not during hypoxia. The Hct in fish fed control and *Ulva* diets at recovery was significantly reduced when compared with hypoxic values, in contrast with the group fed *Gracilaria* diet, where values for this parameter still remained high during recovery. This suggests that dietary modulation by *Gracilaria* may stimulate erythrocyte release and/or formation, although further research needs to address this possible outcome.

The current study also showed that sea bream exposed to acute hypoxia responded by increasing lactate and cortisol concentrations in plasma. This response is in accordance with the general acute stress response described for teleosts, which involves both hypothalamic-sympathetic-chromaffin cell (HSC) and hypothalamic-pituitary-interrenal (HPI) axes activations (Wendelaar Bonga, 1997). Interestingly, the cortisol levels are not only significantly altered by changes in environmental O_2_ conditions, but also were significantly affected by the diet, displaying an interaction between both factors. In fish, lactate accumulation during hypoxia is associated with an increase in reliance on anaerobic metabolism, as a result of HSC and HPI axis activation, with the subsequent release of the stress hormones epinephrine, norepinephrine and cortisol (Vianen et al., 2001). In most fish species, including sea bream, the increase in cortisol levels is reached approximately 1 h after exposure to an acute stressor, with a recovery phase of up to 48 h following, in which cortisol level reach baseline values (Arends et al., 1999; Mommsen et al., 1999; Rotllant et al., 2001). Therefore, it is possible that differences in cortisol levels observed in the dietary groups may be more conspicuous at a shorter sampling time. Although methodological limitations prevented us from sampling fish at an earlier time (e.g. 1 h after hypoxia), the expected release and effect of catecholamines synthesized by the HSC axis may not be ruled out. Thus, this may be an interesting issue to be addressed in future studies on the modulatory effects of cortisol and catecholamines released by the diet formulation. Cortisol has a key role for genomic signalling in the molecular reprogramming in fish tissues, particularly the liver, which is critical for coping with stress. As such, cortisol release may influence the transcriptional regulation of genes involved in energy use and antioxidant response by binding to glucocorticoid receptors (Aluru and Vijayan, 2007).

### Enzymatic markers related to the oxidative stress response in liver of sea bream indicate a modulatory role of dietary SW

Previous studies in common carp *Cyprinus carpio* (Lushchak et al., 2005), rotan *Perccottus glenii* (Lushchak and Bagnyukova, 2007), and medaka *Oryzias latipes* (Oehlers et al., 2007) showed that oxidative stress can be induced by hypoxia. All these results are in line with the animal preparation for coping to oxidative stress conditions, a mechanism proposed by Hermes-Lima et al. (2015), and in accordance with the results presented in our study. Although the mechanisms of hypoxia-induced oxidative stress have not been clearly established yet, it may be possible that a reduction in the mitochondrial electron-transport chain efficiency may contribute to ROS generation (Lushchak, 2011).

Lipid peroxidation is a well-established marker for oxidative tissue damage, and a good indicator of oxidative stress (Gutteridge, 1995). Several factors, including diet and environmental conditions, have an impact on the level of lipid peroxidation detected in fish tissues (Di Giulio et al., 1989; Winston and Di Giulio, 1991). It is known that dietary amino acids (Pérez-Jiménez et al., 2012b; Sitjà-Bobadilla et al., 2005) and fatty acid profiles (Pérez-Sánchez et al., 2013; Saera-Vila et al., 2009) modulate the antioxidant response of sea bream, particularly when subjected to environmental stressors, including hypoxia. In the current study, changes in hepatic markers related to oxidative stress indicate a differential response of sea bream to variations in environmental O_2_ condition. A protective role of SW supplementation is suggested since a decrease in hepatic lipid peroxidation was detected in those fish fed dietary SW-supplemented diets, but not in fish fed the control diet, particularly during recovery. This protective role is reinforced by the differential survival rate observed during hypoxia/recovery of sea bream fed SW-supplemented diets when compared to fish fed the control diet. Additional support for this observation is provided by a low CAT activity detected in liver of sea bream fed the *Gracilaria* diet and exposed to hypoxic condition. CAT activity, mainly associated with peroxisomes, is linked to the protection of the liver from the elevated concentrations of hydrogen peroxide (H_2_O_2_) generated by ROS (Di Giulio et al., 1989; Winston and Di Giulio, 1991). The role of CAT in the protection against ROS formation is displayed by the high activity of this enzyme found in the liver of sea bream during hypoxia, particularly in the group fed the control diet.

GPX and GR are enzymes involved in the antioxidant response to environmental stress in aquatic organisms (Winston, 1991; Winston and Di Giulio, 1991). GPX function is to reduce lipid hydro-peroxides to alcohols generated by ROS (Di Giulio et al., 1989). In parallel, the function of GR is to re-establish antioxidant capacity (Di Giulio et al., 1989). The current study showed that the activity of GR and GPX hepatic enzymes were altered in response to environmental O_2_ fluctuations, with GPX activity showing a significant reduction in the *Gracilaria* group during hypoxia. In summary, our results suggested an imbalance between prooxidant/antioxidant in liver of sea bream, displayed by changes in the activities of several enzymes linked to oxidative stress in response to changes in the environmental O_2_ condition, a response that was shown to be diet-modulated.

### The transcriptional responses in heart and liver of sea bream indicate dietary SW supplementation takes a protective role

The hypoxia switch from OXPHOS to anaerobic glycolysis results in reduced mitochondria O_2_ consumption and enhanced NADH production from glycolysis (Frezza et al., 2011). Furthermore, experimental evidence indicates that the gene expression of enzyme subunits of the mitochondrial respiratory chain is highly regulated in a tissue-specific manner by the type and intensity of environmental stressor in gilthead sea bream (Bermejo-Nogales et al., 2014). In the present study, the OXPHOS ratio, taken as the index of Complex I (primary electron donor) versus Complex IV (final O_2_ acceptor), was higher in aerobic cardiac muscle than in liver. Importantly, the same trend was even more evident when comparing normoxia versus recovery in both liver and heart. The OXPHOS ratio could reflect the importance of mitochondrial activity and respiration, whereas the overall balance among encoded catalytic subunits (ND2, ND5, COXI, COXII) or even other enzymatic complexes can provide an estimation of changes in the respiratory chain efficiency (Theron et al., 2000). Thus, an increased OXPHOS ratio could be indicative of improved capacity to aerobically generate energy surplus after a hypoxic episode, allowing a greater electron transport through mitochondria when O_2_ concentrations are restored. Citrate synthase (CS) is a marker of mitochondrial abundance (Larsen et al., 2012; Rabøl et al., 2009) and a measure of the Krebs cycle capacity, and the enzyme function is coordinated with the respiratory chain enzyme activities (Holloszy et al., 1970). The decreased FA oxidation/mitochondrial abundance ratio during recovery highlights an enhancement of aerobic capacity mediated by the greater relative importance of CS in relation to CPT1A (mitochondrial FA-carrier), as noted by the lower CT values (higher mRNA abundance) in CS gene with almost no variation in CPT1A during recovery (data not shown). This fact may indicate that the acetyl-CoA produced by β-oxidation of FAs is rapidly used during hypoxia recovery to feed the Krebs cycle and to produce NADH and FADH2, which in turn are used by the OXPHOS pathway to permit a more efficient ATP production from aerobic respiration. However, the increased reliance in the use of substrates produced during hypoxia, such as lactate, could not be ruled out and may also explain the minor importance of the aerobic fatty acid oxidation pathway during recovery. In this scenario of hypoxia resilience and recovery, both OXPHOS and CPT1A/CS indexes were regulated in a tissue-specific manner by O_2_ availability, but not by dietary treatment. In contrast, a number of markers, including transcription factors, antioxidant and oxidative enzymes, molecular chaperones and mitochondrial uncoupling proteins were differentially regulated by dietary supplementation of heat-treated *Gracilaria* and *Ulva* during normoxia and/or recovery as explained below for some of these differentially expressed genes.

HIF-1α, a well-described regulator of the adaptive response of fish to changes in environmental O_2_ availability, is known to up-regulate the expression of antioxidant enzymes in response to oxidative stress (Lushchak and Bagnyukova, 2006). In our study, the expression of this transcription factor was down-regulated during recovery of the hypoxic episode in both liver and heart of fish fed the *Gracilaria* diet. This finding might be interpreted as a steady state of minimized risk of oxidative damage during tissue reoxygenation, which is perhaps a direct consequence of an improved and more efficient response to hypoxia exposure. In heart, and to a lesser extent in liver, this observation was also supported by the concurrent down-regulation of some down-stream markers of cell redox balance and oxidative stress, including UCP1, antioxidant defence enzymes (GPX4, PRDX3, and PRDX5) and molecular chaperones (GRP-75, GRP-94). However, the down-regulation of HIF-1 expression in both tissues should be interpreted cautiously, as this transcriptional factor is mostly post-transcriptionally regulated. Uncoupling proteins (UCPs) are mitochondrial transporters that uncouple OXPHOS attenuating the production of ROS (Rial and Zardoya, 2009). Previous work in sea bream showed that hepatic UCP1 expression is altered by a wide range of stressors including confinement and winter cold exposure (Bermejo-Nogales et al., 2010). GPX4 is a well-described seleno-peroxidase of vertebrates with a major protective role in oxidative damage, which inhibits lipid peroxidation by reducing H_2_O_2_ and complex membrane lipid hydroperoxides (Thomas et al., 1990). GRP-75, also known as mortalin or mitochondrial HSP70, has been shown to be a stress biomarker in sea bream, with induced hepatic expression in response to crowding stress and parasite *Enteromyxum leei* infections (Bermejo-Nogales et al., 2008; Calduch-Giner et al., 2012). In a similar way, antioxidant enzymes such as GPX4, PRDX3, PRDX5, and the molecular chaperone GRP-94 were also identified as highly stress-responsive elements in crowded fish with different stress responsiveness according to their nutritional background (Saera-Vila et al., 2009). Thus, the observed down-regulation of all these factors in sea bream fed the *Gracilaria*-extract diet reinforces the idea of a protective effect of the heat-treated SW supplementation with better recovery after hypoxia, as compounds with antioxidant properties present in the supplemented dietary extract may reduce the requirement for antioxidative enzymes.

In fish fed the heat-treated *Ulva* diet, the expression profile of differentially expressed genes in the liver tissue also showed a marked down-regulation of antioxidant-related markers during normoxia. However, this molecular signature was not maintained during the recovery stage, which suggests a lower capacity to counteract the triggered ROS production that occurs during hypoxia in comparison with the *Gracilaria* group (Guzy and Schumacker, 2006). Conversely, the heart of fish fed heat-treated *Ulva* diet experienced a down-regulation of antioxidant markers (ECH, PRDX3, GRP-94, GRP-75) during recovery, but again the number of differentially expressed markers and the magnitude of the fold-change variation was lower than in the *Gracilaria* group. It is also important to note that in both groups fed heat-treated SW diets the heart showed a more marked response than liver tissue in terms of the number of differentially expressed genes during recovery. This feature is not surprising, since sea bream cardiac muscle was previously reported in a microarray study to be the most responsive to nutrient restriction when compared with skeletal white and red muscle (Calduch-Giner et al., 2014). This probably reflects the high metabolic plasticity of heart as a tissue that must be highly regulated to maintain its essential functions. In this context, heart emerges as a highly promising target tissue for stress responsiveness.

In both SW groups, the transcriptomic results are in accordance with those of enzyme activity of CAT, GR, GPX. The experimental evidence of the improved survival rate found in *Gracilaria* or *Ulva* groups is conclusive to the beneficial effect of SW supplementation in gilthead sea bream diet. Therefore, results observed in this study are in accordance with the potential protective role of heat-treated *Gracilaria* against oxidative stress. Compounds with antioxidant properties present in this SW extract may reduce the requirement of antioxidative enzymes, decrease oxidative damage in tissues, and fish mortalities produced by changes in environmental O_2_ condition. However, this study did not evaluate antioxidant content in the SW extracts. Future studies will be required to identify compounds contained in heat-treated *Gracilaria* and to clarify possible mechanisms involved in the antioxidant capacity of sea bream other than the glutathione system. Differences in the dietary-induced response observed in *Gracilaria* and *Ulva* groups may be related to the different compounds produced and contained in the extracts of these red or green SW, respectively. It has been described that the antioxidant effect of dietary SW, including *Gracilaria sp.*, may be related to the presence of polyphenols in their composition (Ganesan et al., 2008; Jiménez-Escrig et al., 2001; Sachindra et al., 2010; Sreenivasan and Ibrahim, 2007), as they may break up free-radical chains of oxidation and donate hydrogen (Duh, 1998). The antioxidant properties of *Gracilaria sp.* could be linked to their sulphated polysaccharide content as well (Costa et al., 2010; Guaratini et al., 2012; Qi et al., 2005; Souza et al., 2012), as those compounds may act as an electron donors to minimize the attack of free radicals (Fidelis et al., 2014). On the other hand, compounds with antioxidant properties which may be included in the *Ulva* diet (e.g. polysaccharides, phenols or flavonoids) appear to be less effective in the *Gracilaria* diet, due to differences in the type, quantity or changes produced by the extraction of such compounds (Reverter et al., 2014; Stengel et al., 2011). The practical implications of this research for the aquaculture industry are clear since the susceptibility of aquatic organisms to biotic and abiotic stressors is becoming a restrictive factor in fish produced in intensive aquaculture conditions.

### Conclusions

The dietary modulation of the oxidative stress responsiveness in gilthead sea bream by heat-treated SW was less conspicuous under normoxia, but became more evident during and after the hypoxic challenge. The physiological response and survival rate of gilthead sea bream to changes in environmental O_2_ condition was highly modulated by the inclusion of heat-treated SW in the diet. The observed changes in oxidative stress response reveal the beneficial effects of the dietary SW supplementation in sea bream, particularly when *Gracilaria vermiculophylla* is included in the diet. Both SW diets also altered oxidative stress response in liver and heart by down-regulating the gene expression of different antioxidant enzymes and molecular chaperones during recovery. This study suggests that compounds with antioxidant properties present in the supplemented dietary extract reduce the requirement for antioxidative enzymes.

## MATERIALS AND METHODS

### Animal care and rearing conditions

All procedures were conducted under the supervision of an accredited expert in laboratory animal science by the Portuguese Veterinary Authority (1005/92, DGV-Portugal, following FELASA category C recommendations), according to the guidelines on the protection of animals used for scientific purposes from the European directive 2010/63/UE. The experiment took place at the Abel Salazar Biomedical Sciences Institute (ICBAS), University of Porto (Portugal). This study was approved by the ORBEA (Organismo Responsável pelo Bem-Estar dos Animais), the Institutional Animal Care and Use Committee (IACUC) of the ICBAS. Fish were anesthetized with MS-222 ethyl 3-aminobenzoate methanesulfonate (MS-222, 0.1 g l^−1^), buffered with NaHCO_3_ (0.2 g l^−1^) for blood collection, and subsequently sacrificed by decapitation.

Sexually immature gilthead sea bream was provided by IPMA (Instituto Português do Mar e da Atmosfera, Olhão, Portugal) and reared in recirculation tanks for acclimation for at least three months before the trial. During that time fish were fed with a commercial diet (SPAROS, Portugal) at a maintenance ration with a photoperiod regime that was artificially regulated into an automatic 12 h:12 h (day:night cycle). Two weeks prior to starting the trial, sea bream were distributed over 24 experimental tanks (60 l) connected to a water recirculation system at a flow rate of 250 l h^−1^. Each experimental tank contained seven fish with an average weight of 104.5 g (11.65 kg m^−3^). Temperature (19.0°C), salinity (38‰), pH (8) and dissolved O_2_ (above 95% saturation) were fixed and monitored daily, and regulated whenever necessary.

### Diets

*G. vermiculophylla* and *U. lactuca*, were produced in land-based IMTA (Integrated Multitrophic Aquaculture) systems by ALGAPLUS Lda. (Ilhavo, Portugal). Dried *G. vermiculophylla* was thermally processed using hot water at 83°C for 160 min. The mixture was filtered with a cloth and the agar was recovered through a freeze-thawing process. The solid product from the thermal process was washed, dehydrated with ethanol and dried at 60°C overnight under vacuum. *U. lactuca* was thermally processed using hot water at 89°C for 152 min. The heat-treated product was filtered and the solid residue was dried overnight at 60°C under vacuum.

The control diet and two SW diets (5% Gracilaria, 5% Ulva) were formulated and manufactured by SPAROS Lda. (Olhão, Portugal). Diet formulation and chemical composition are presented in Table S2. Powder ingredients, including the heat-treated SW, were ground (<100 micron) in a micropulverizer hammer mill (Hosokawa Micron, SH1, The Netherlands). Ingredients were then mixed according to the target formulation in a paddle mixer (Mainca RM90, Spain) and the mixture was humidified with 25% water. Diets were cold extruded (below 60°C, pellet size: 2.0 mm) by means of a low shear extruder (Italplast P55, Italy). Upon extrusion, all feed batches were dried in a convection oven (OP 750-UF, LTE Scientifics, UK) for 3 h at 45°C.

### Experimental procedure

Tanks were assigned either: (i) a diet without SW (control); (ii) a diet supplemented with 5% *Gracilaria*; or (iii) a diet with 5% *Ulva* using a randomized block design. Fish were hand-fed two meals per day (09:30 and 16:30 h) for 34 days to apparent satiety. A known quantity of diet was weighed daily in excess of estimated feed intake and placed in 24 goblets, one for each tank, and gradually fed to fish. The cessation of feeding was decided by visual observation of uneaten pellets within 15 min. Uneaten pellets in the tank were subsequently collected, counted and the total weight was estimated by taking into account the average weight of the dry pellets. The actual daily feed intake per tank was then calculated by subtracting the initial feed in the goblet by the total leftover feed. No mortality was observed during the feeding trial.

**Fig. 5.**
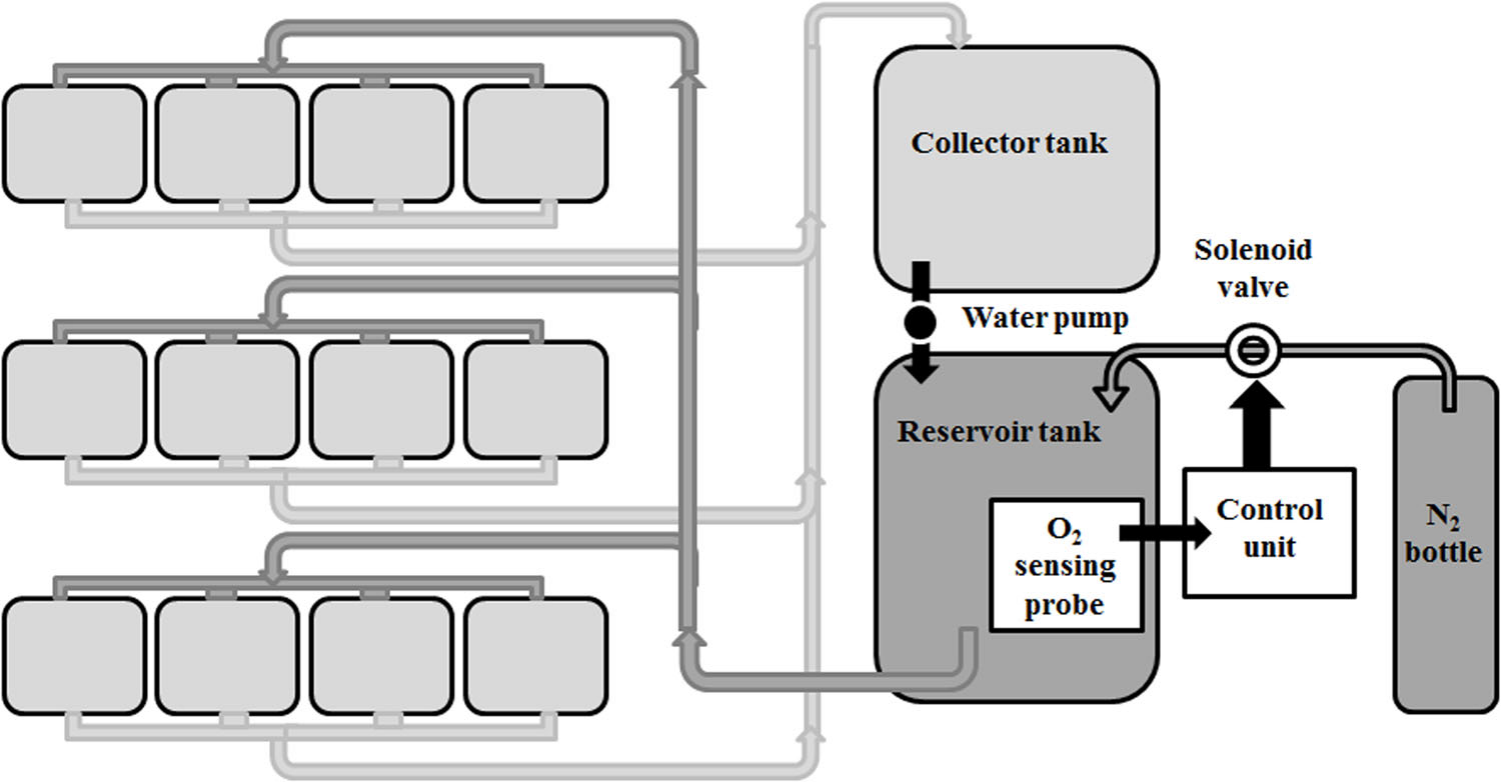
**Experimental set-up used to control dissolved oxygen (DO) levels in the experimental tanks to implement hypoxia.** The system consisted of a loop of 12 tanks with reduced DO levels, which were separated from the remaining tanks (12) in normoxia as detailed in the Materials and Methods section.

**Fig. 6.**
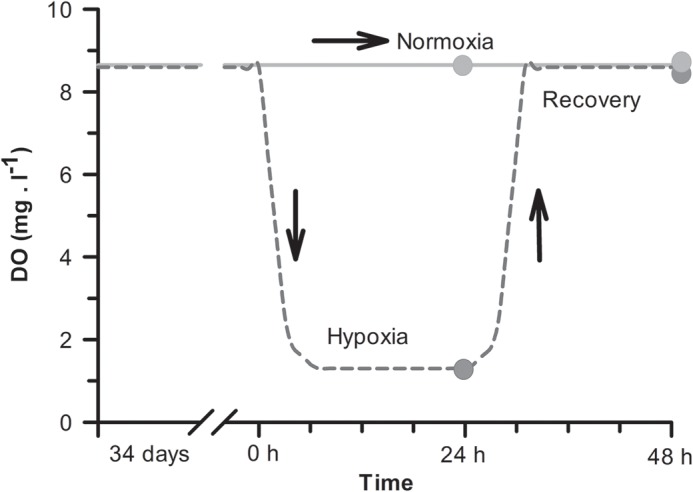
**Changes in dissolved oxygen (DO) levels during the trial.** Sea bream fed the experimental diets for 34 days were subjected to normoxia (8.6 mg O_2_ l^−1^) or to hypoxia (1.3 mg O_2_ l^−1^) followed by normoxia (recovery) as described in the Materials and Methods section. Sampling points for each experimental condition are represented as dots.

At the end of the feeding trial, 24 h fasting fish from 12 tanks (*n*=4 tanks/dietary treatment) were subjected to a 24 h acute hypoxic condition (1.3 mg O_2_ l^−1^), returning to normoxia (8.6 mg O_2_ l^−1^) thereafter. The remaining 12 tanks (*n*=4 tanks/dietary treatment) remained under normoxic conditions during the entire trial. Fish were subjected to an acute severe hypoxia set to reach 17.5% O_2_ saturation, below the limiting O_2_ saturation value (LOS or Pcrit) reported for this species at 31% O_2_ saturation at 19°C (Remen et al., 2015). The low dissolved O_2_ levels were obtained by using controlled injection of nitrogen gas into a reservoir tank following a similar setup as described by Behrens and Steffensen (2007). On this method, the nitrogen influx into the system was continuously monitored and controlled by an O_2_ analyser and regulator system (OXY-REG, Loligo Systems, Denmark) equipped with a galvanic O_2_ probe (mini-DO, Loligo Systems, Denmark, range 0-200% air saturation) using a negative feedback loop regulating a solenoid valve (Loligo Systems, Denmark) connected to a nitrogen bottle (see Fig. 5). The O_2_ probe was calibrated according to manufacturer's instructions. The surface of the water in the hypoxic tanks was covered completely by polycarbonate panels to minimize air exchange. The dissolved O_2_ levels were gradually reduced in the 12 hypoxic tanks as shown in Fig. 6. Twenty-four hours after initiating the hypoxia, dissolved O_2_ levels in the tanks were gradually returned to normoxia (recovery). Mortality was directly assessed by visual observation and defined as the point when the opercular movement ceased in fish at 15 h and 24 h of hypoxia, as well as during recovery (24 h after cessation of hypoxia challenge).

### Fish sampling

Four to eight fish per treatment were randomly selected and sampled immediately at 24 h of hypoxia (hypoxia) and after 24 h returning to normoxia (recovery). Similar sampling procedures were used for fish that remained in normoxic condition throughout the entire trial (normoxia). Fish were anaesthetized with MS-222 (0.1 g l^−1^), buffered with NaHCO_3_ (0.2 g l^−1^), and then weighed. Blood was collected from the caudal vein using syringes containing EDTA (0.5 M, pH 8, GIBCO) and plasma was obtained (5 min, 10.000 rpm at 4°C) and stored at −80°C, until analysed. After sacrificing the fish by decapitation, heart and liver samples were immediately dissected and frozen in liquid nitrogen. Samples, stored at −80°C, were transported either to CIIMAR (Porto, Portugal) to assess several stress markers or shipped to IATS-CSIC (Institute of Aquaculture Torre de la Sal, Spain) to perform transcriptomic analyses. Growth performance parameters were calculated at the end of the experiment.

### Blood and plasma analyses

Haemoglobin concentration in blood samples was measured using a kit for quantitative colorimetric determination (Drabkin, 1001231, SPINREACT, Sant Esteve de Bas, Spain). Lactate concentration in plasma was quantified using a commercial kit (UV method, AK00131, NZYTech, Lisbon, Portugal). Cortisol in plasma samples was quantified using an enzyme immunoassay (Cortisol ELISA kit, RE52061, IBL International, Hamburg, Germany). All measurements were performed in triplicates, following the recommendations provided by the manufacturers.

### Oxidative stress markers in liver

Liver samples were homogenized in phosphate buffer (1/10 vol., 0.1 M pH 7.4). Enzymatic analyses were all carried out with the reaction mixtures and homogenate dilution established in preliminary tests. Protein concentration was assayed in homogenates using bovine serum albumin as standard (Bradford, 1976). LPO was determined by quantifying the presence of thiobarbituric acid reactive substances (Ohkawa et al., 1979). Catalase (EC 1.11.1.6.) activity was analyzed with hydrogen peroxide (30%) as substrate (Clairborne, 1985). GR (EC1.8.1.7) and GPX (EC 1.11.1.9.) were evaluated based on NADPH (Sigma, Portugal) oxidation at 340 nm (Cribb et al., 1989; Mohandas et al., 1984). Glutathione s-transferase (GST) (EC 2.5.1.18) was determined using 1-chloro-2,4-dinitrobenzene as substrate (Habig et al., 1974). Changes in absorption were measured at 22°C in a Power-Wave™ microplate spectrophotometer (BioTek Instruments), and reactions were performed in triplicates. Substrate was omitted in controls and background activity was subtracted from that measured in the presence of substrate.

### Gene expression analyses

Total RNA from liver and heart was extracted using a MagMax-96 total RNA isolation kit (Life Technologies, Carlsbad, CA, USA). RNA concentrations were obtained with UV absorbance measures (A260/280) and RNA quality was determined using an Agilent 2100 Bioanalyzer (Agilent Technologies). RIN (RNA Integrity Number) values were 8-10, for almost all samples, which was indicative of clean and intact RNA to be used in quantitative real-time PCR (qPCR) reactions.

Synthesis of cDNA was performed with the High-Capacity cDNA Archive Kit (Applied Biosystems, Foster City, CA, USA) using random decamers and 500 ng of total RNA in a final volume of 100 µl. Reverse transcriptase (RT) reactions were incubated 10 min at 25°C and 2 h at 37°C. Negative control reactions were run without RT. qPCR reactions were performed using an Mastercycler^®^epgradient S Realplex2 with Realplex software v.2.2 (Eppendorf, Hamburg, Germany). Diluted RT reactions were conveniently used for qPCR assays in 25 µl volume in combination with a SYBR Green Master Mix (Bio-Rad, Hercules, CA, USA) and specific primers at a final concentration of 0.9 µM (Table S1). The 96-well PCR-array layout was designed for the simultaneous profiling of a panel of 27 genes, related to oxidative metabolism and oxygen sensing, antioxidant defense, xenobiotic metabolism, cellular stress response (molecular chaperones) and growth-promoting action (GH/IGF system) (Table S6). The program used for PCR amplification included an initial denaturation step at 95°C for 3 min, followed by 40 cycles of denaturation for 15 s at 95°C and annealing/extension for 60 s at 60°C. All the pipetting operations were made by means of an EpMotion 5070 Liquid Handling Robot (Eppendorf, Hamburg, Germany) to improve data reproducibility. The efficiency of PCRs (>92%) was checked, and the specificity of reactions was verified by analysis of melting curves (ramping rates of 0.5°C 10 s^−1^ over a temperature range of 55-95°C) and linearity of serial dilutions of RT reactions (>0.99). Fluorescence data acquired during the extension phase was normalized by the delta-delta Ct method (Livak and Schmittgen, 2001) using *β-actin* (Actb) as the housekeeping gene. For multi-gene analysis, data on gene expression was in reference to the expression levels of ECH in fish fed the control diet, for which a value of 1 was arbitrarily assigned in each tissue and environmental O_2_ condition.

The range of variation for Ct values of comparisons of Actb was lower than 0.25-0.40 cycles among dietary treatments for a given environmental O_2_ condition. Nevertheless, this range was increased up to 0.40-1.10 cycles between normoxia and recovery. Thus, in order to compare different environmental O_2_ conditions, two different algorithms (with no dependance of housekeeping gene uniformity) related to OXPHOS ([ND2+ND5]/[COXI+COXII]) and fatty acid oxidation/mitochondria abundance (CPT1A/CS) were proposed as time-course and tissue-specific markers of oxidative capacity under different environmental O_2_ conditions.

### Calculations and statistical analyses

Zootechnical parameters were calculated using the tank as the experimental unit (*n*=8). Weight gain (WG, %) was calculated as:

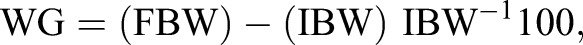


where FBW is the average final body weight (g), and IBW is the average initial body weight (g).

Feed conversion ratio (FCR) was calculated as:

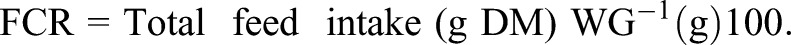


The accumulated mortality in the hypoxia/recovery groups was calculated as the percentage of dead fish per tank (*n*=4), averaging values per dietary treatment over specific times. Mean corpuscular haemoglobin concentration was calculated as follows:

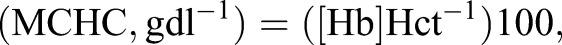


where [Hb] is the concentration of haemoglobin in blood (g dl^−1^) and Hct the hematocrit value (%).

For analysis and calculations of physiological parameters, the fish were used as the experimental unit with a sample size of five to seven per experimental treatment. However, given that blood and plasma parameters, as well as enzymatic activities, were not significantly different (*P*>0.05) between fish sampled at 24 h or 48 h of normoxia for a given dietary treatment (see Fig. 6), values for both groups were pooled and expressed as normoxic values (*n*=14). Values are presented as means±s.e.m. For biochemical and enzymatic parameters, a two-way ANOVA analysis was used for making comparisons between treatments, and a Holm-Sidak post hoc analysis was used to identify significant differences between treatments.

Changes in relative gene expression for a given tissue and dietary treatment were analysed by one-way ANOVA followed by the Holm-Sidak test, or by Kruskal–Wallis H test followed by Dunn's method, in each case. Fold-changes of mRNA expression levels in differentially expressed genes fed under different dietary treatments were analysed with respect to the control group by Student *t*-test. The significance level was set at *P*<0.05. All analyses were performed using the SigmaPlot Version 13 for Windows.
